# Evaluation of a Web-Based Wellness Program to Reduce Loneliness Among Older Adults

**DOI:** 10.1093/geroni/igab046.3311

**Published:** 2021-12-17

**Authors:** Rifky Tkatch, Lizi Wu, Laurie Albright, Charlotte Yeh

**Affiliations:** 1 UnitedHealth Group, Oak Park, Michigan, United States; 2 UnitedHealth Group, Minnetonka, Minnesota, United States; 3 UnitedHealth Group, Minneapolis, Minnesota, United States; 4 AARP Services, Inc., Washington, District of Columbia, United States

## Abstract

Background: Loneliness has been associated with adverse health outcomes, including increased mortality risk. Interventions aimed at addressing maladaptive social cognition have documented efficacy in reducing loneliness among older adults. Purpose: The purpose of this study was to determine the feasibility and efficacy of a web-based wellness program in reducing loneliness and improving psychosocial health among older adults with an AARP® Medicare Supplement plan insured by UnitedHealthcare Insurance Company. Methods: Eligible individuals were 65 years and older, who self-identified as lonely on a prior survey, and indicated that they had access to the internet through a computer or smartphone. Participants completed up to eight online modules comprised of a short lesson on an aspect of maladaptive social cognition, followed by a comprehension quiz, selection of a short-term goal, and a phone or text chat with a program coach. Four surveys were administered to assess the effects of program participation: (1) prior to the start of the program, (2) after completion of four online modules, (3) after completion of all eight modules, and (4) 30-60 days later. Results: Attrition was high. Overall, 220 (42%) program participants completed both T1/T2 surveys, 193 also completed a T3 survey, and 177 also completed a T4 survey. Post-survey data indicated that loneliness and perceived stress decreased while mental wellbeing, resilience, and perception of aging improved. Conclusion: Digital interventions aimed at addressing maladaptive social cognition offer potential to reach lonely older adults and support psychosocial wellbeing through reduced loneliness.

